# Autoimmune Glial Fibrillary Acidic Protein (GFAP) Astrocytopathy and Its Radiological Manifestations: A Case Report

**DOI:** 10.7759/cureus.104137

**Published:** 2026-02-23

**Authors:** Azwade Rahman, Avraham Y Bluestone

**Affiliations:** 1 Diagnostic Radiology, Stony Brook University, Stony Brook, USA; 2 Radiology: Neuroradiology, Stony Brook University Hospital, Stony Brook, USA

**Keywords:** astrocytopathy, autoimmune glial fibrillary acid protein astrocytopathy gfap, autoimmun encephalitis, gfap, glial fibrillary acidic protein, helminth infection, meningo-encephalitis, microglia and astrocyte dysfunction, neuroradiology, toxic exposure

## Abstract

Neuronal astrocytes rely on a cytoskeletal element known as glial fibrillary acidic protein (GFAP), which facilitates their main structural framework, maintaining neurological function, repair, and lastly the blood-brain barrier. Pathologies such as neoplasms, autoimmune-mediated inflammation, and even genetic dysfunction can directly harm this element and result in malfunctioning astrocytes, CNS inflammation, and cell death. In this case report, we will create a timeline from presentation to remission of a rare entity known as autoimmune GFAP astrocytopathy for a middle-aged male with a wide variety of exposures and concomitant conditions. This case is unique, as in many cases, only one antibody marker was found on CSF analysis. After careful review of imaging from our institutional PACS, select slices were anonymized and organized here for review. The study aims to highlight the timeline of imaging features and their evolution. Advanced imaging techniques were also present and contributed to previous supporting evidence. Prior studies describe this entity with a wide variety of imaging features. Its most unique feature may be radial perivascular enhancement. What was not previously known is when these findings may present and how their absence within the time period of imaging may impact the investigation.

## Introduction

Autoimmune astrocytopathy targeting the intermediate cytoskeleton is a disease that occurs so infrequently that there are not enough cases to establish a definitive epidemiology. It was first acknowledged under the name of glial fibrillary acidic protein (GFAP) astrocytopathy in 2016 [1]. Since its recognition, there have been many thoughts on the gaps in the literature on this disease. What is known is that up to a third of cases are related to paraneoplastic syndrome. The most frequently associated primary tumor is ovarian teratoma, though it should be noted that there is no gender predilection as per current studies on the topic. Other neoplasms associated with this condition include gliomas, adenocarcinomas, squamous cell carcinomas of the head and neck, and multiple myeloma. Other proposed triggers to condition include Epstein-Barr virus infection, traumatic brain injury, concomitant autoimmune disorders, herpes simplex virus encephalitis, dengue, syphilis, and COVID-19 infection [1]. Encephalitis can induce a bimodal pattern in which the first peak of symptomology occurs during the viral invasion, with a following second peak from the autoantibodies. It can occur at any age but has a reported median age of 44-50 years. Pediatric patients account for 10% of the total cases [2-4]. A fifth of patents are also found to have coexisting autoimmune disorders [5,6]. These may include type 1 diabetes, autoimmune thyroiditis, or rheumatoid arthritis.

The cytoskeletal element is a major component of astrocytes within the central nervous system. It helps provide their structure as well as facilitates their function, such as ensuring the integrity of the blood-brain barrier and healing. These elements can be found in Schwann cells and glial cells within the enteric nervous system. The pathophysiology of attacking this system is not clearly delineated; however, there is a known clinical presentation that can be divided into meningoencephalitis, which accounts 55% of presentations, and meningoencephalomyelitis, which accounts for 40% of presentations [7]. The first scenario can include symptoms such as altered mental status, headaches, meningeal symptoms, nausea, tremors, vision changes, seizures, and secondary psychiatric manifestations. The second scenario adds sensorimotor dysfunction. Optic neuritis, optic disc edema, and disc papillitis are ocular manifestations of this condition, with bilateral disc edema being the most common. This symptomology is nonspecific and can be present in a litany of conditions on the autoimmune spectrum. The most common mimics are acute disseminated encephalomyelitis (ADEM), myelin oligodendrocyte glycoprotein antibody-associated disease (MOGAD), and neuromyelitis optic spectrum disorder (NMO) [1]. Part of the gap in knowledge in the literature is with regard to the inconsistencies in the imaging pattern, which may cause delays in care and require unnecessary workup.

Among all that is known, there is very little consensus on the distinguishing radiological findings regarding this disease. Also, there is no set temporal progression of the disease related to its identification and treatment. The gold standard is CSF analysis demonstrating GFAP-IgG. The imaging features of this entity are a helpful segue toward this diagnosis beyond clinical assessment. According to past studies, imaging manifestations on magnetic resonance imaging can range from 44% to 66% of total cases [5,7,8]. Fluid-sensitive imaging shows hyperintense lesions involving the periventricular white matter. This can also involve the basal ganglia, centrum semiovale, limbic cortex, and extend inferiorly toward the brainstem [3]. However, the most classical feature from the limited available cases is linear perivascular radial enhancement perpendicular to the ventricles in the white matter. This feature is present in 44% of cases [6]. This indicates areas of severe perivascular inflammation. The morphology of the enhancement may also vary, including a punctate to nodular appearance. It may also include leptomeningeal, serpentine, or ependymal distributions. Cranial nerves are also at risk of involvement. The structures of the posterior fossa are much less frequently impacted. The susceptibility of weighted imaging tends to be negative for metallic deposition or hemorrhage. Unfortunately, many of these imaging features may also be mimicked by the same conditions that are noted under different clinical findings. ADEM, NMOSD, and MOGAD are included in this differential, but chronic lymphocytic inflammation with pontine perivascular enhancement responsive to steroids (CLIPPERS) and supratentorial lymphocytic inflammation with parenchymal perivascular enhancement responsive to steroids (SLIPPERS) may also provide this presentation [1]. 

This clinical case will give an overview of a case of GFAP autoimmune astrocytopathy from presentation, diagnosis, and treatment, to follow-up. The step-by-step overview hopes to give more insight into the evolution of this rare entity and more temporal characteristics of its previously described key radiologic features. Clinical and imaging correlations are important in helping guide decision-making in its progression. What this case adds to the literature is supportive evidence of positive and negative findings, including the utilization of advanced techniques such as MR perfusion and MR spectroscopy. Further, we discuss how presentation and imaging mimics can impact appropriate and directed therapy. The value of this case is also the unique aspect of documenting the temporal evolution of GFAP astrocytopathy through imaging and different techniques. The hope is to avoid unnecessary treatment and investigation following the points presented here. This may also contribute to the development of optimal timing for initial and repeat imaging in order to gauge treatment response and/or disease progression. The pertinent findings of this case may also contribute to the development of imaging protocols in investigating these entities. 

## Case presentation

A 45-year-old Caucasian male with obesity, hypertension, and gout presented to the emergency department with concern for an acute ischemic infarct. He was experiencing worsening left-sided weakness and was unsteady for five days prior to presentation, leaning more to the left as he walked. His wife also noticed that the patient's speech was slowing and he appeared more confused. He had a facial droop on the left side of his face, a slight left arm drift, weakness in grip strength, as well as weakness in the left leg in hip flexion and plantar flexion, according to the ED physicians. Bell’s Palsy was recorded in his past medical history. The history of gout is further complicated by the patient undergoing helminthic exposure through a naturopathic provider. The patient was suspected of having GI colonization of* Necator americanus* by the naturopathic helminthic therapy.

**Figure 1:**
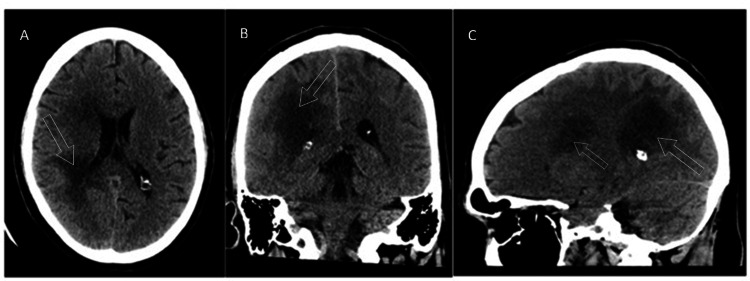
Initial nonenhanced CT Unenhanced CT head showing decreased attenuation of the right cerebral white matter, as designated by the unfilled white arrows. The findings were primarily concentrated within the parietal but also were seen in the occipital, posterior temporal, and frontal white matter within the axial (A), coronal (B), and saggital (C) planes. There was an associated mass effect as suggested by the sulcal and lateral ventricular effacement.

**Figure 2:**
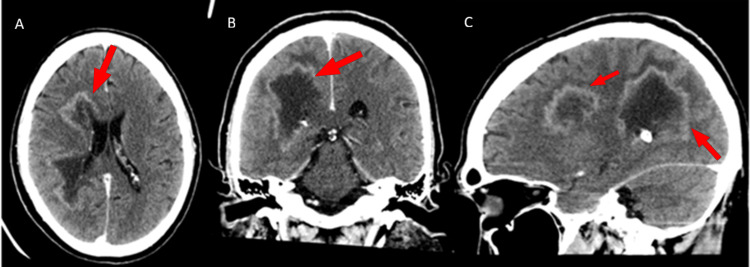
Postcontrast CT on presentation On postcontrast imaging, the lesion demonstrated peripheral enhancement in the involved regions as shown in the axial (A), coronal (B), and sagittal (C) planes. The enhancement pattern is highlighted by the red arrows.

His labs were significant for hypokalemia, measuring up to 3.0 mEq/L. His drug screen was positive for both cocaine and cannabinoids. There was a trace elevation in his white cell count, measuring 12.9 mm^3^. A non-enhanced head CT is shown in Figure 1, with a follow-up post-contrast study shown in Figure 2. The reading radiologist assessed that the lesion was also compressing the right middle cerebral artery (MCA) branches.

**Figure 3:**
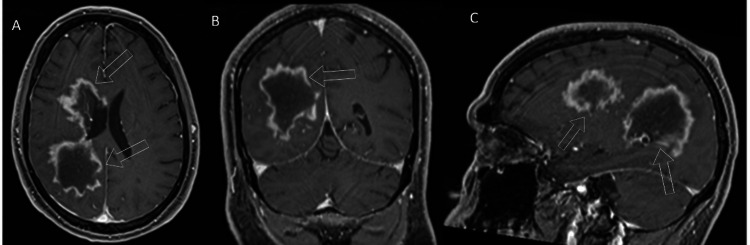
Initial MRI on presentation Enhanced MRI demonstrates mass-like lesions with a T1 hypointense signal relative to unaffected white matter with avid peripheral enhancement as marked by the unfilled white arrows. The provided views included axial (A), coronal (B), and sagittal (C). The enhancement pattern is frondlike and slightly discontinuous.

The follow-up MRI did not demonstrate diffusion restriction to suggest an infarct. Instead, there was a mass-like T1 hypointense/T2 hyperintense lesion predominantly situated at the right parietal lobe with peripheral enhancement (Figure 3). Not shown here is a peripheral high signal on diffusion weighted imaging with associated low apparent diffusion coefficient (ADC) values consistent with reduced diffusivity. More defined on the MR, the enhancement pattern showed frondlike extensions into the corona radiated with small areas of discontinuity.

**Figure 4:**
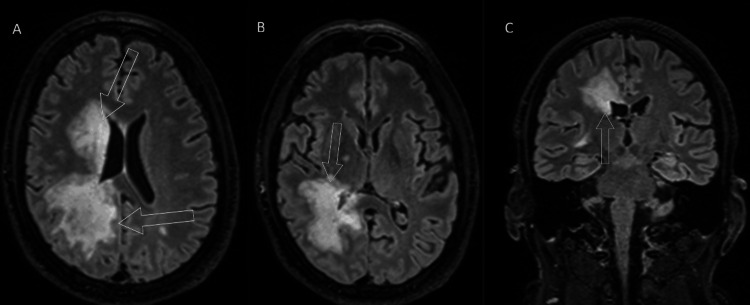
Same-day MRI T2 FLAIR sequences T2 fluid-attenuated inversion recovery (FLAIR) sequences confirm the distribution of the affected brain, as marked by the unfilled white arrows. Two axial slices at the level of the lateral ventricles (A) and the basal ganglia (B) are provided. For further illustration, a single coronal view of the third ventricle (C) is also shown. What is also apparent now is the involvement of the right basal ganglia, as depicted in the second provided slice.

There was no apparent leptomeningeal enhancement or brainstem involvement. T2 fluid-attenuated inversion recovery (FLAIR) sequences confirmed this distribution, as shown in Figure 4.

The T2 FLAIR showed a larger confluent area of affected parenchyma, which was not as readily apparent on the initial postcontrast CT. These findings initially raised the suspicion for a neoplastic process, and as such, a follow-up full-body CT was administered. No findings indicative of a primary tumor or metastatic disease were evident.

Enhanced MR of the spine also failed to demonstrate significant findings aside from a focal area of central canal prominence at the C6-C7 level, which is a normal variant.

**Figure 5:**
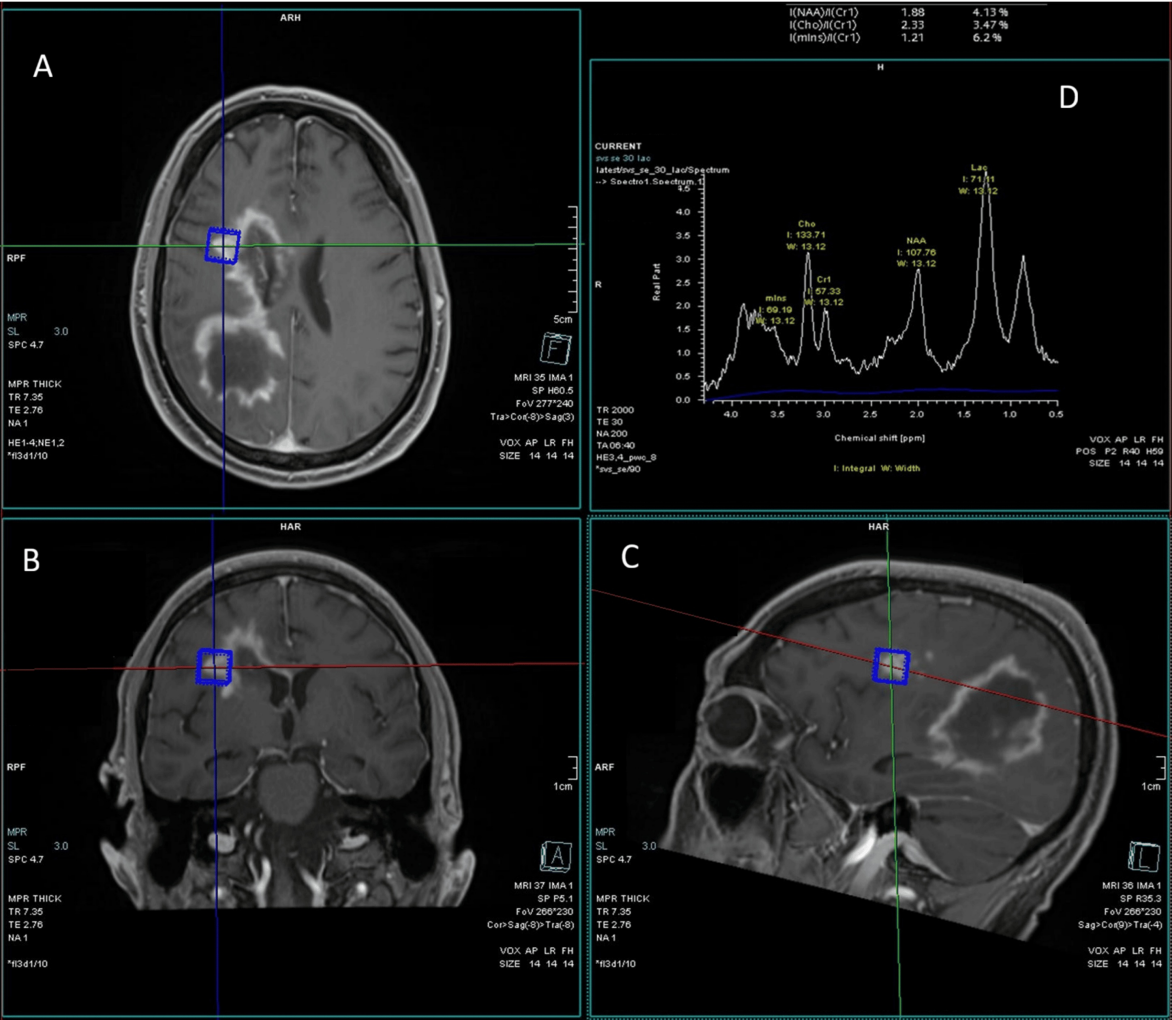
MR spectroscopy MR spectroscopy demonstrates elevated choline and lactate within the enhancing portions of the lesion. The *N*-acetylaspartate peak was decreased. Not shown is that the spectroscopy directed at the nonenhancing portions of the lesions showed marked lactate peaks. The selected area of interest is depicted in the axial (A), coronal (B), and saggital (C) planes. The graphical analysis is shown in image D with the values shown as 4.13% for N-acetylaspartate (NAA), 3.47% for choline, and 6.2% for creatinine.

Follow-up spectroscopy imaging with an echo time of 30 ms (Figure 5) showed elevated choline and lactate peaks at the enhancing portion of the lesions with an associated decreased NAA peak. Not shown is that the corresponding nonenhancing portions showed marked lactate peaks.

**Figure 6:**
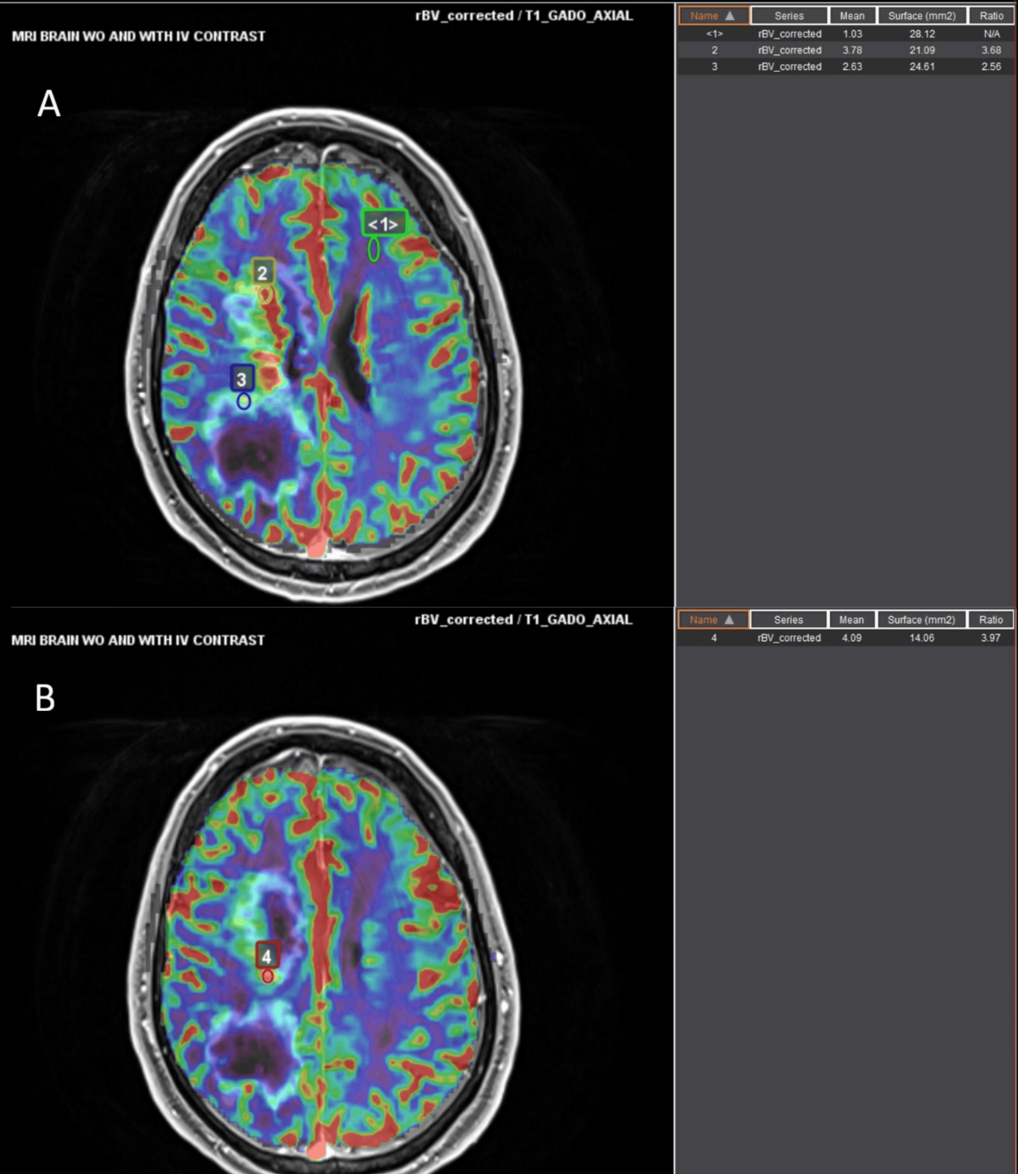
MR DSC perfusion Dynamic susceptibility contrast (DSC) MR perfusion applied to the original MRI brain demonstrated elevated relative cerebral blood volume within the enhancing margins of the lesions. Image A shows points 1-3, which correspond to relative cerebral blood volume (rCBV) values of 1.03, 3.78, and 2.63, respectively. Image B shows point 4, which corresponds to an rCBV value of 4.09.

A dynamic susceptibility contrast MR perfusion analysis was also applied to the original MRI brain, which showed an elevated relative cerebral blood volume within the enhancing portions of the lesions, estimated to be 4.1 times relative to the uninvolved white matter (Figure 6). 

At this point, on imaging, the differential included tumefactive demyelination, infectious leukoencephalopathy, and toxic/metabolic leukoencephalopathy, though necrotic neoplasm could not be excluded. Lumbar puncture CSF analysis was conducted and demonstrated a markedly elevated protein of 122.7 mg/dL, glucose of 78 mg/dL, and trace nucleated cells without pleocytosis. The patient began steroid therapy with 10 mg Decadron daily. Video electroencephalogram (EEG) showed a hemispheric asymmetry with continuous focal slowing over the right hemisphere, suggestive of focal cerebral dysfunction. Infectious workups remained negative. 

Follow-up flow cytometry discovered the key findings of oligoclonal bands, an elevated immunoglobulin G (IgG) index, and elevated proteins. These findings made the differential more supportive of a neuroinflammatory disorder and prompted continued corticosteroid therapy with follow-up in two to four weeks. The consulted neurosurgery team also opted against a brain biopsy. From an infectious disease standpoint, there was persistent suspicion for JC virus, toxoplasmosis, *Tropheryma whipplei*, and HIV. 

**Figure 7:**
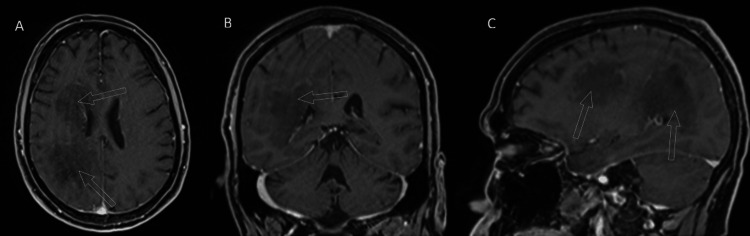
First MRI following treatment Repeat imaging following corticosteroid therapy showed near-complete resolution of the peripherally enhancing lesion of the right parietal lobe, with the areas of interest marked by the unfilled arrow. The axial (A), coronal (B), and sagittal (C) planes are displayed. There is still evidence of persistent mass effect as suggested by effacement of the right lateral ventricle.

Repeat MR imaging was obtained a little over a week from admission and following high-dose steroid therapy, as displayed in Figure 7.

**Figure 8:**
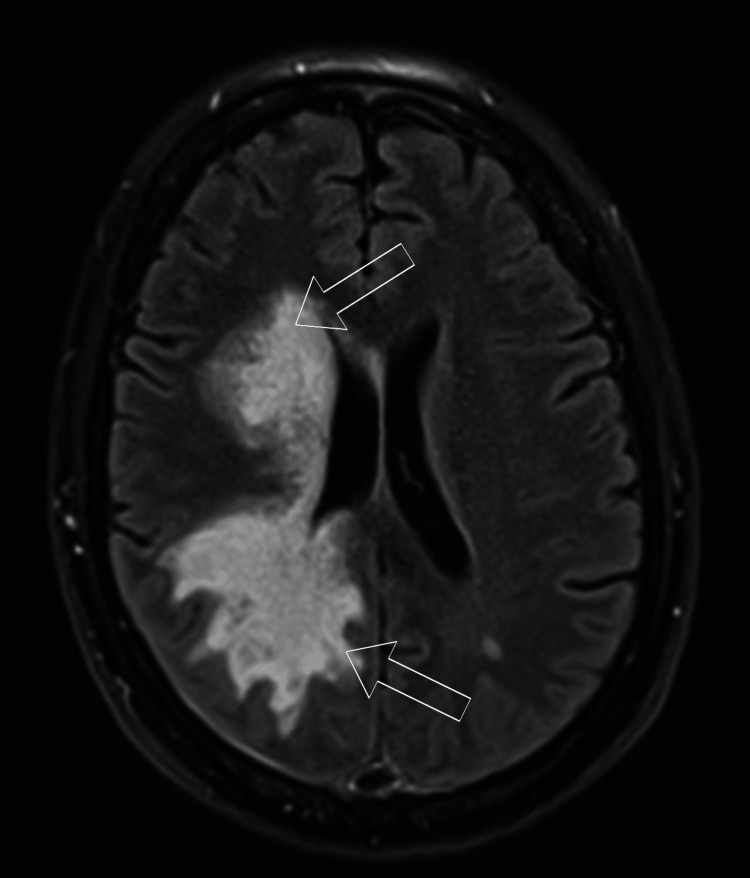
Follow-up post-treatment T2 FLAIR sequence T2 fluid-attenuated inversion recovery (FLAIR) sequence of repeat MR demonstrating a persistent signal in the associated areas as depicted by the unfilled arrows.

While the enhancement and T1 hypointensity of the lesion had largely resolved at that point, there was still significant edema within the region, as suggested by the T2 FLAIR signal findings demonstrated in Figure 8.

The MR perfusion analysis on this study demonstrated only foci of hyperperfusion remaining within the affected areas in comparison to the normal white matter. Clinically, the patient reported improvement in his asymmetrical weakness.

**Figure 9:**
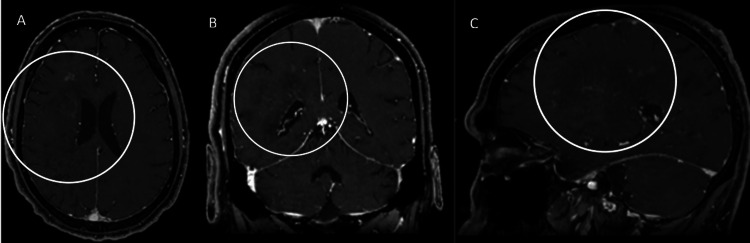
Radial periventricular enhancement on follow-up Follow-up enhanced MR of the patient demonstrated faint radial periventricular enhancement, as opposed to the predominantly peripheral enhancement that was seen on priors. The affected areas are marked by unfilled white circles on the axial (A), coronal (B), and sagittal (C) images.

Nearly a month following his initial admission, repeat imaging was obtained, and a new pattern of enhancement seemed to emerge. The results are shown in Figure 9.

**Figure 10:**
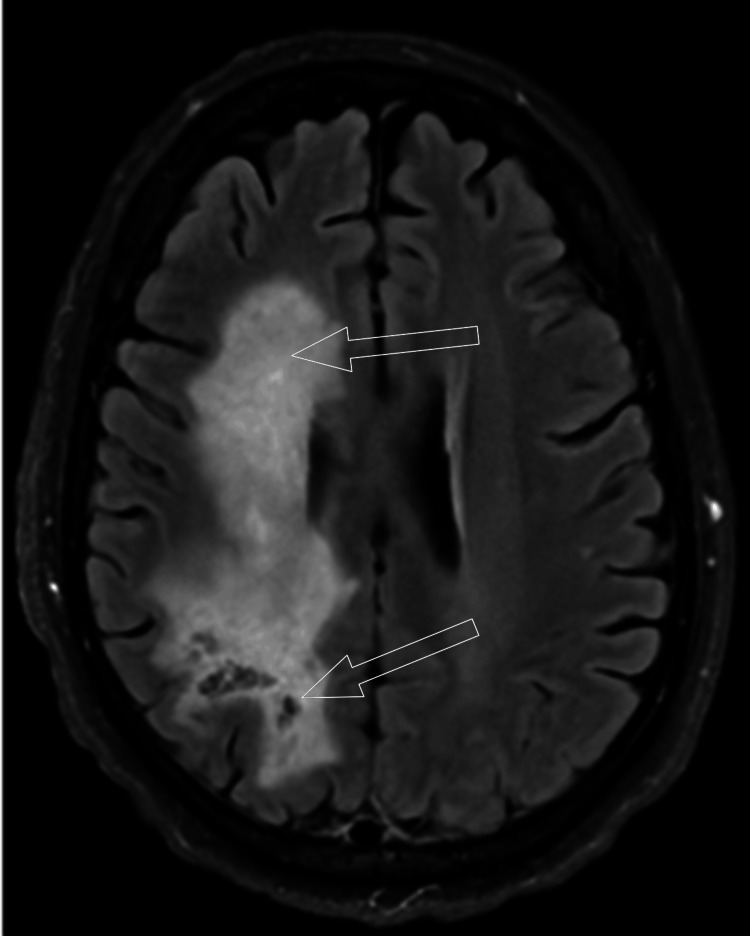
Continued evolving on FLAIR Post-treatment changes on the T2 fluid-attenuated inversion recovery (FLAIR) sequences. The caudal unfilled arrows demonstrate signal loss consistent with gliosis as opposed to the frontal arrow, which shows a persistent, uniform abnormal signal.

Now, faint radial enhancement appeared to emerge as treatment progressed, reminiscent of the working diagnosis. The FLAIR sequence also evolved, as displayed in Figure 10.

The T2 FLAIR signal continued to grow; new areas of signal loss were also noted. It was also around this time that outside CSF analysis demonstrated a reactive result to GFAP immunofluorescence assay (IFA) as well as a positive GFAP cell-based assay (CBA), definitively pointing toward the diagnosis of autoimmune GFAP astrocytopathy. The titer was positive with a dilution ratio of 1:16. No other positive findings could be reported. As such, steroid therapy was continued, and follow-up was continued into the outpatient setting.

**Figure 11:**
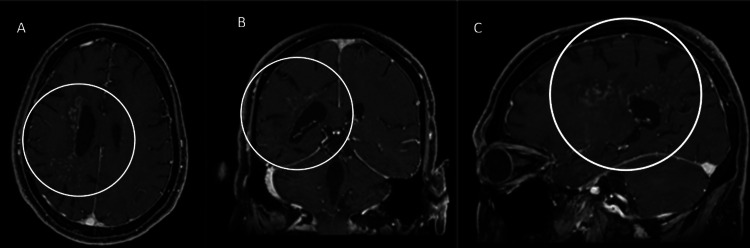
Five-month follow-up enhanced MRI At five months post-treatment, the patient's enhancement patterns show curvilinear and nodular enhancement speckled throughout the white matter. It predominates in a perivascular and periventricular distribution. The axial (A), coronal (B), and sagittal (C) planes are depicted with the abnormal enhancement marked by the unfilled circles.

After nearly five months of steroid therapy, the patient's symptoms began to improve. Previously wheelchair bound due to the immense deficits caused by his condition, the patient was now able to ambulate. The working diagnosis became immune-mediated GFAP astrocytopathy. The patient was trialed on a steroid taper. Follow-up imaging is shown in Figure 11.

**Figure 12:**
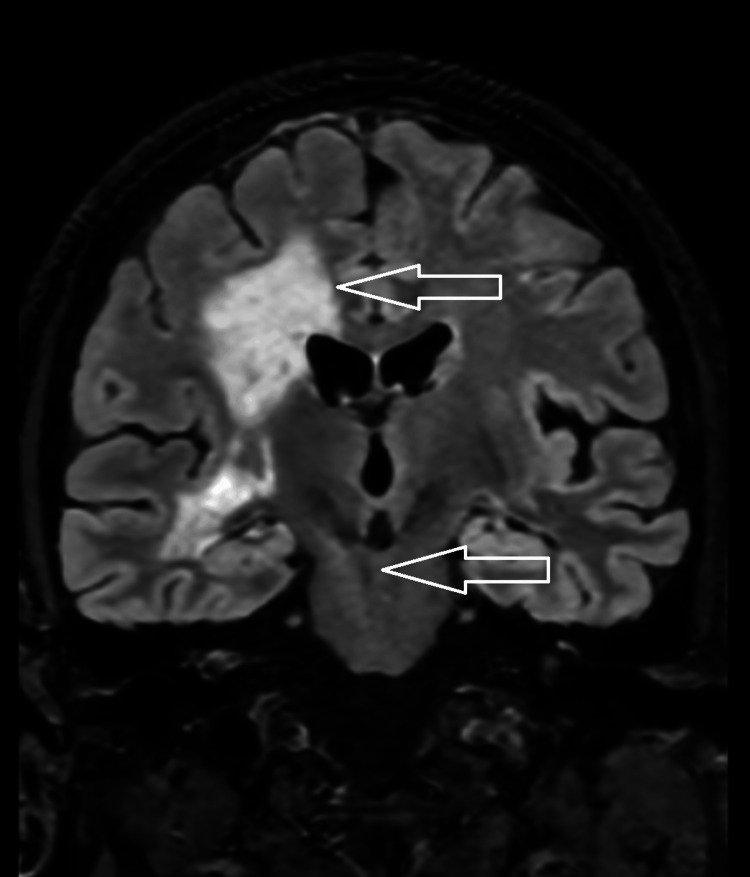
FLAIR signal with descending spread Coronal T2 fluid-attenuated inversion recovery (FLAIR) showing involvement largely within the right cerebral white matter as depicted by the superior unfilled arrow. A newly conspicuous FLAIR hyperintensity is seen within the right midbrain and pons, pointed out by the inferior unfilled arrow.

The resurgence of the enhancement following the steroid taper suggested progression. Furthermore, there was evidence of extension of the affected brain, as there was a signal extending into the right cerebral peduncle and pons on the T2 FLAIR sequence, which is demonstrated in Figure 12. Interestingly, there was notable peripheral diffusion restriction at the right basal ganglia on the patient's MRI. Although not shown, this finding was weakly persistent five months following initial presentation.

## Discussion

The patient initially presented with weakness in a pattern that was concerning for infarction; however, the pattern of diffusion restriction did not support this diagnosis, and a wider differential needed to be explored. The presented imaging data, as well as CSF analysis, revealed itself to be an extremely rare case of autoimmune GFAP astrocytopathy. What is especially helpful in this case is the fact that the CSF analysis showed pure findings of antibodies unique to autoimmune GFAP astrocytopathy without other antibodies, as was seen in prior literature. The subject case falls in line with other CSF analysis findings, in which 80% of patients demonstrated elevated proteins [5]. Though there was only trace pleocytosis, which is a feature found in up to 78.5% of patients [5]. Correlation with past studies on histological analysis cannot be conducted, as the subject patient did not undergo a biopsy. As such, it provides a unique opportunity to characterize our findings through the lens of a single existing entity. This case is presented with a wide differential that is manifested in both clinical and radiographic findings. During the investigation, vascular, malignant, and infectious pathologies were all evaluated concomitantly with immunological etiologies. Ultimately, the robust response to high-dose steroid therapy, as well as the analysis of the cerebrospinal fluid on top of the evolving imaging findings, is what clinched the diagnosis.

**Figure 13:**
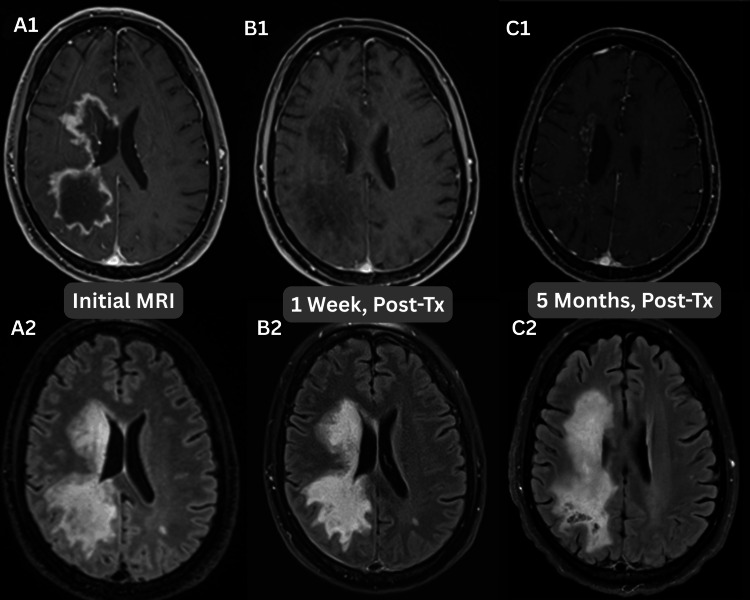
MRI post-contrast and T2 FLAIR Axial slice images at the level of the lesion at different time points, presenting on both T1-weighted and T2 fluid-attenuated inversion recovery (FLAIR) sequences. Images A1 and A2 are from the initial presentation, B1 and B2 are from one week post-treatment, and C1 and C2 are from five months post-treatment.

Initially, the imaging shows a mass-like hypodensity within the right white matter tracts and associated with the basal ganglia, which corresponded to mass-like T1 hypointense/T2 hyperintense lesions with peripheral enhancement. The lesions had frondlike extensions with mass effect against the local structures. These findings mimicked subacute infarction, necrotic neoplasm, tumefactive demyelination, and toxic/metabolic leukoencephalopathy. These findings have been seen in prior literature. GFAP astrocytopathy can share characteristics with multiple inflammatory and demyelinating disorders, including NMOSD, MOGAD, ADEM, and CNS infections such as JC virus or toxoplasmosis [1]. An interesting point in this case is the evolution of imaging findings over time. The classic radial enhancement pattern did not become apparent until five months of post-treatment with steroid therapy. These findings are depicted in Figure 13.

**Figure 14:**
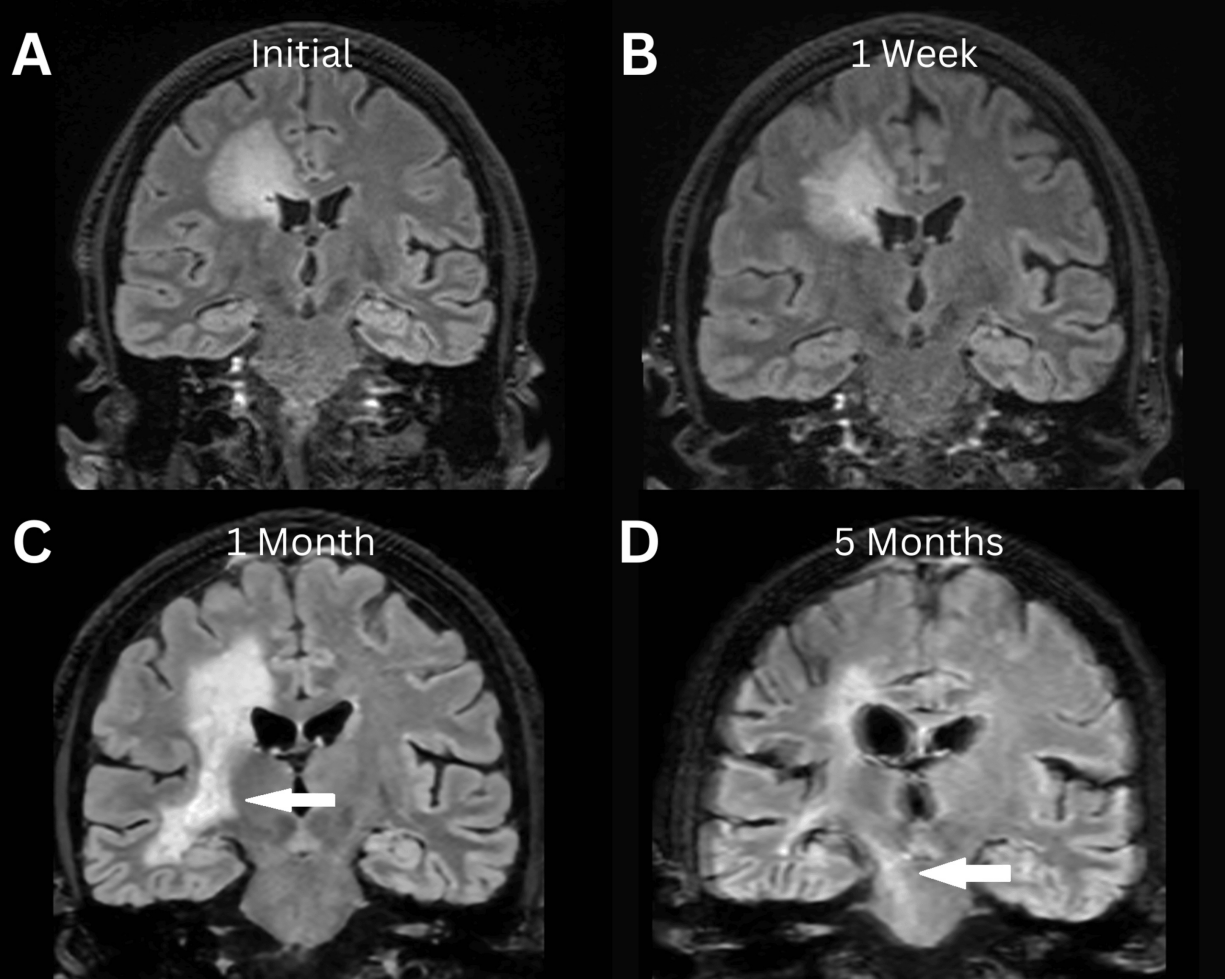
Spread of T2 FLAIR signal to the brain stem On coronal views, the T2 fluid-attenuated inversion recovery (FLAIR) signal can be seen extending from the periventricular white matter and descending toward the brain stem at different time points from the initial presentation. (A) One week after presentation, (B) One month after presentation, and (C) Five months after presentation. (D) White arrows in images C and D show the new areas of involvement over time.

Spinal findings were not present in our case, as was present in prior literature in 49% of cases [7]. Among those, 71% had cervical spine involvement, 65% had thoracic involvement, and 23% had conus or cauda equina involvement. However, brainstem pathology was observed in 34% of patients. What is interesting is that the brainstem involvement did not present on imaging until further along in the patient's course, as well as within the posttreatment phase of the disease. The gradual evolution of this involvement is depicted in Figure 14. 

These findings on images may show further on the temporal evolution of this disease and may advise clinicians not to exclude it from the differential diagnosis based on imaging if they are not present at the time point following presentation. The mechanism behind this evolution is something to investigate further. The true pathophysiology of this condition is unknown. The autoantibodies targeting the cytoskeleton are thought to be biomarkers within the CSF and serum, which are a result of the cytotoxic T-cell-mediated immunity [4]. These antibodies cannot attack intracellular components on their own. As such, there is a proposed mechanism of a triggering event that exposes the intracytoplasmic GFAP to the immune system to induce the propagation of the antibodies [3]. As briefly touched upon within the introduction, viral stigmata, trauma, or other autoimmune processes are suspected of inciting the aberrant process. CSF studies may also present cytotoxic T-cells, which support the idea of cellular response being the centerpiece of pathophysiology. One of the key features that was present in this case is the radial perivascular enhancement, which in the subject case is presented in a speckled pattern throughout the white matter. Histologically, there is significant perivascular cellular infiltration from immunological actors [8]. This corresponds with what is known about the mechanism of action of the disease leading up to its radiological manifestations.

Interestingly, within their literature, there are cases in which CSF analysis found autoantibodies to NMDA, AQ4, and antineuronal nuclear antibodies, which support a preceding autoimmune component of the disease [9]. This patient had a past medical history of gout, which was associated with cytotoxic T-cell-mediated cellular response [10]. This may be a new hypothesis that was not previously described in the literature. Another interesting point regarding this was the naturopathic therapy the patient had chosen to undergo for his gout. Helminthic therapy has been described previously as a path to immunomodulation to control inflammatory conditions [11]; however, it has also been known to cause overactivation of the immune system [12]. This may be a new, potential hypothesis to explore regarding a trigger.

Toxic exposure, as suggested by the patient’s cocaine use, is also an interesting thought of an inciting factor, as it has been associated with increased cytokine production and lymphocyte changes [13]. The potential of toxic exposure as a trigger should also be considered for further study.

A prior case described in prior literature discussed a 64-year-old male with a six-month history of symptoms, which differs from our case, which presented after less than a month [14]. It suggests that this hallmark pattern may be a stage of its temporal evolution, preceded by a lesion with a tumefactive appearance. Within this case, MR spectroscopy was also used to analyze their subject. The results demonstrated an abnormal increase in the choline peak with a decrease in the N-acetylaspartate peak [14]. This almost exactly mirrors the findings in the present case, with the exception of a lactate peak. Our imaging findings hope to build upon the evidence of the findings on advanced imaging techniques.

The remarkable improvement in response to steroid therapy was consistent with the concluded diagnosis [15]. Of note, there was a resurgence of edema following the tapering of steroid therapy. Relapse is a feature of this disease that has been previously described, and as touched upon previously, may be associated with a viral infection. The relapse involved a greater extent of the white matter, extending to the level of the brainstem. This emphasizes the importance of clinical monitoring and follow-up during the treatment phases. Serial imaging, CSF analysis, and treatment response were critical in establishing the correct diagnosis and avoiding unnecessary biopsy. This case hopes to provide some insight into a protocol for monitoring what features to look for at certain intervals. Clinicians should consider GFAP astrocytopathy in subacute presentations with evolving white-matter lesions and steroid responsiveness, even in the absence of classical patterns.

## Conclusions

The importance of this case highlights how many neurological symptoms and imaging can mimic a wide variety of pathologies, and careful monitoring and serial imaging can help direct care efficiently and effectively. The evolution of a lesion on imaging may completely change the course of investigation. Prior literature mainly discusses the presence of certain imaging features of autoimmune GFAP astrocytopathy, but it fails to highlight how these findings may appear at certain time points within the disease. The understanding of this disease based on time may contribute to its diagnostic criteria on imaging. CSF analysis remains the gold standard; however, it may not always be the case, as the presence of other markers may distract from the primary entity. The role of radiology is to be aware of the differentials and unique features, such as radial enhancement, which may not present on initial imaging. After GFAP autoimmune astrocytopathy is successfully identified, this case demonstrates the need for careful monitoring following the evolution of treatment plans, as relapse can occur frequently. Being open to these possibilities can prevent unnecessary, invasive, and expensive investigations. The variable exposures in this case also provide potential future directions of investigation in future cases.
